# First-line immune checkpoint inhibitors in older adults (≥75 years) with advanced esophageal squamous cell carcinoma: efficacy and safety

**DOI:** 10.3389/fonc.2025.1753874

**Published:** 2026-01-14

**Authors:** Lin-lin Zheng, Yu-cai Jiang, Dan-dan Lin

**Affiliations:** 1 Department of Oncology, Affiliated Hospital of Putian University, Putian, Fujian, China; 2 Department of Pharmacy, Affiliated Hospital of Putian University, Putian, Fujian, China; 3 Department of Research, Affiliated Hospital of Putian University, Putian, Fujian, China

**Keywords:** 75 years and older, advanced esophageal squamous cell carcinoma, antineoplastic agents, efficacy, immunotherapy

## Abstract

**Background:**

The combination of immune checkpoint inhibitors with chemotherapy is a standard first-line treatment for advanced esophageal squamous cell carcinoma. However, robust data regarding the efficacy and safety of this approach specifically in patients aged 75 years and older are scarce, as this population is frequently underrepresented in pivotal clinical trials.

**Objective:**

This study aimed to evaluate the real-world treatment outcomes and safety profile of first-line immunotherapy in patients aged ≥75 years with advanced esophageal squamous cell carcinoma.

**Methods:**

We conducted a retrospective, single-center cohort study. Sixty-one patients aged ≥75 years with histologically confirmed, advanced esophageal squamous cell carcinoma (stage III/IV) who initiated first-line immune checkpoint inhibitors (as monotherapy or combined with chemotherapy/antiangiogenic therapy) between February 2021 and May 2025 were included. Assessments included tumor response per RECIST 1.1, progression-free survival, overall survival, and adverse events graded per the National Cancer Institute Common Toxicity Criteria v5.0.

**Results:**

The median age was 80 years. The objective response rate was 45.9% (28/61), and the disease control rate was 85.2%. After a median follow-up of 16.7 months, the median progression-free survival was 12.7 months (95% CI: 6.9-17.1), and the median overall survival was 17.7 months (95% CI: 10.7-22.8). Both progression-free survival and overall survival significantly differed among response groups (log-rank *p* < 0.0001). Treatment was generally tolerable. Any-grade immune-related adverse events occurred in 39.1% of patients, with grade ≥3 events in 1.6%. Non-immune toxicities were common (any grade 93.1%; grade ≥3 in 14.7%), primarily hematologic.

**Conclusion:**

In this real-world cohort, first-line immunotherapy demonstrated promising efficacy and a manageable safety profile in patients aged ≥75 years with advanced esophageal squamous cell carcinoma. These findings provide valuable preliminary evidence supporting the use of immune checkpoint inhibitors in this understudied population and highlight the need for prospective validation to optimize therapeutic strategies.

## 1 Introduction

Esophageal cancer is one of the leading causes of cancer-related death worldwide, with an estimated 511,000 new cases and 445,000 deaths reported in 2022 (1). It ranks as the 11th most commonly diagnosed cancer and the seventh leading cause of cancer mortality globally (2). More than half of these incident cases and deaths are estimated to occur in China (3, 4). The prognosis for esophageal squamous cell carcinoma (ESCC) remains poor, as the majority of patients are diagnosed at advanced stages, resulting in a 5-year survival rate of only 10-30% (5, 6). The use of immune checkpoint inhibitor represents a breakthrough in the treatment of advanced ESCC. Blockade of programmed cell death-1 (PD-1) and its ligand (PD-L1) resulted in improved overall survival (OS) in the first-line and second-line settings (7, 8). Subsequent phase 3 studies showed that the addition of immune checkpoint inhibitor to chemotherapy resulted in longer OS and progression-free survival (PFS) than chemotherapy alone (9–13).

However, the evidence base derived from pivotal randomized trials remains limited regarding very elderly patients. Older adults, particularly those aged ≥75 years, are frequently underrepresented or excluded from registration and large phase III studies, leaving clinicians with sparse high-level data on the efficacy and tolerability of first-line immune checkpoint inhibitor (ICI)-based regimens in this growing patient subgroup (14–16). Physiological changes associated with aging, a higher prevalence of comorbidities, altered pharmacokinetics, and increased vulnerability to treatment-related toxicity collectively raise critical concerns regarding the risk–benefit balance when considering intensive combination regimens in older adults. Although pooled analyses and real-world evidence across tumor types suggest that elderly patients may derive clinical benefit from immune checkpoint inhibitors (ICIs) without substantially higher rates of severe immune-related adverse events (irAEs) compared with younger populations, data specific to patients aged ≥75 years with advanced esophageal squamous cell carcinoma (ESCC) remain limited and highly heterogeneous (17, 18).

Given these knowledge gaps, there is an urgent need for studies specifically evaluating first-line ICI-based therapies in patients aged ≥75 years with advanced ESCC to guide clinical decision-making. In particular, real-world evidence on tumor response, survival outcomes, and the spectrum of treatment-related adverse events in this elderly population can enable clinicians to more accurately weigh potential benefits against risks and tailor treatment intensity accordingly. This retrospective, single-center cohort study was therefore conducted to assess the efficacy and safety of first-line ICI-based regimens in older patients (≥75 years) with advanced ESCC, aiming to generate clinically meaningful data to inform individualized therapeutic strategies in this vulnerable and underrepresented population.

## 2 Materials and methods

### 2.1 Study design and patients

A retrospective analysis was performed at the Affiliated Hospital of Putian University, China, involving patients aged 75 years or older with advanced esophageal squamous cell carcinoma (stage III/IV). Eligible participants were those who initiated first-line immunotherapy between February 2021 and May 2025.

The inclusion criteria were as follows: (1) Patients diagnosed with ESCC according to the diagnostic criteria outlined by the World Health Organization, confirmed by imaging and pathological examination, and aged ≥ 75 years; (2) Patients staged as III or IV using the TNM classification; (3) Patients with complete medical records; (4) Patients who underwent the specific drug treatment and had post-treatment outcomes evaluated; (5) at least one measurable lesion according to Response Evaluation Criteria in Solid Tumors, version 1.1 (RECIST v1.1).

**Figure 1:**
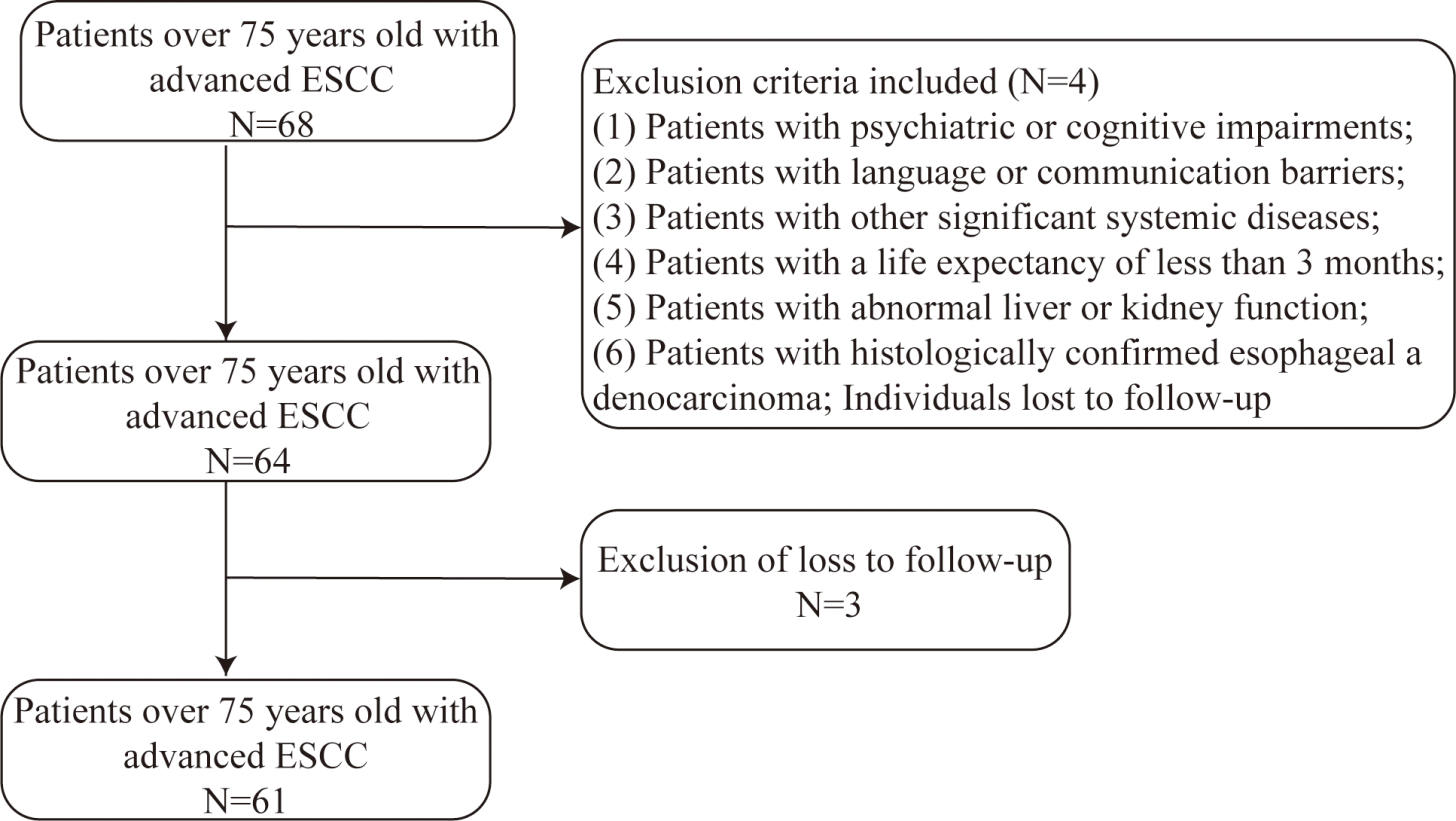
Patient flowchart detailing the inclusion and exclusion criteria for the retrospective cohort of patients aged ≥75 years with advanced esophageal squamous cell carcinoma treated with first-line immunotherapy.

The exclusion criteria encompassed: (1) Patients with psychiatric or cognitive impairments; (2) Patients with language or communication barriers; (3) Patients with other significant systemic diseases; (4) Patients with a life expectancy of less than 3 months; (5) Patients with abnormal liver or kidney function; (6) Patients with histologically confirmed esophageal adenocarcinoma; Individuals lost to follow-up (**Figure 1**).

The chemotherapy regimen consisted of albumin-bound paclitaxel combined with carboplatin. Chemotherapy dosing was based on body surface area: albumin-bound paclitaxel 200 mg/m^2^ administered intravenously on Day 1, and carboplatin dosed to an area under the concentration–time curve (AUC) of 4 on Day 1. Immune checkpoint inhibitors included sintilimab 200 mg, tislelizumab 200 mg, camrelizumab 200 mg, toripalimab 240 mg, and pembrolizumab 200 mg, each given intravenously on Day 1. All treatments were administered on a 3-week cycle. Computed tomography (CT) scans were performed every 2–3 cycles to assess treatment efficacy.

Criteria for Radiotherapy Selection: The decision for radiotherapy was made following the principles and recommendations outlined in the Chinese Society of Clinical Oncology (CSCO) guidelines for esophageal cancer (19). This included considerations such as palliative intent for symptomatic lesions (e.g., pain, obstruction) and treatment for oligometastatic disease, which guided the selection of the 41% of patients who received radiotherapy.

Imaging Intervals for Response Assessment: We now explicitly state that tumor response was assessed by contrast-enhanced CT scans performed at routine intervals of approximately 6–9 weeks (or earlier if clinically indicated). Adverse Event Grading Definitions: It now clearly states that adverse events (AEs) were graded per CTCAE version 5.0, and CSCO: Management of immune checkpoint inhibitor-related toxicity (20).

The final follow-up was conducted in September 2025. Clinical data extracted from electronic medical records included sex, age, Eastern Cooperative Oncology Group (ECOG) score, Charlson Comorbidity Index, tumor stage, treatment strategies, and sites of distant metastasis. Tumor response was evaluated according to RECIST version 1.1. PD was radiologically confirmed by the treating physician per RECIST 1.1, and that death was ascertained from hospital records or official death registries. Objective response rate (ORR) was defined as the proportion of patients achieving a complete or partial response, and disease control rate (DCR) included patients with complete response, partial response, or stable disease. OS was defined as the interval from immunotherapy initiation to death from any cause or the last follow-up. PFS was defined as the date of immunotherapy to the date of disease progression or death.

### 2.2 Statistical analysis

To analyze survival outcomes in patients aged ≥75 years with advanced esophageal squamous cell carcinoma, Kaplan–Meier curves were plotted, and between-group differences were compared using the log-rank test. Prognostic factors were identified through univariate and multivariate Cox proportional-hazards regression analyses. Corresponding 95% confidence intervals (95% CI) were calculated for all estimates. Statistical significance was defined as a two-sided *p* < 0.05. All analyses were performed using R version 3.3.2 (http://www.R-project.org, The R Foundation) and Free Statistics software version 2.3.

## 3 Results

### 3.1 Clinical characteristics

A total of 61 patients were included in the final analysis. Baseline clinical characteristics are summarized in **Table 1**. The median age was 80 years (range: 75-92). Male patients accounted for 33 (54.1%). Most patients had an ECOG performance status of 0-1 (83.6%) and a Charlson Comorbidity Index (CCI) score of 0 (73.8%). The majority were diagnosed at stage IV (52.5%). Radiotherapy was administered in 25 patients (41.0%). First-line treatment regimens consisted of immune checkpoint inhibitor (ICI) monotherapy in 8 patients (13.1%), ICI combined with anlotinib in 1 patient (1.6%), and ICI plus chemotherapy in 52 patients (85.3%). ICIs used included camrelizumab in 36 patients, tislelizumab in 16, sintilimab in 4, toripalimab in 1, and pembrolizumab in 4. Lymph node metastasis was the most common site of metastasis in advanced ESCC, occurring in 77.0% of cases.

**Table 1:** Baseline clinicopathological characteristics of patients.

Characteristics	Overall (N = 61)
Age, years [Median (IQR)]	80 (75-92)
Gender, n. (%)	
Male	33 (54.1)
Female	28 (45.9)
ECOG PS, n. (%)	
0-1	45 (73.8)
2-3	16 (26.2)
CCI, n. (%)	
0	51(83.6)
1-2	10(16.4)
TNM Stage, n. (%)	
Stage III	29 (47.5)
Stage IV	32 (52.5)
Radiotherapy, n. (%)	
Yes	25 (41)
No	36 (59)
Immunotherapy strategies, n. (%)	
ICI	8 (13.1)
ICI + Anlotinib	1(1.6)
ICI + Chemotherapy	52 (85.3)
Distant metastasis, n. (%)	
Lymph node metastasis	47(77)
Lung metastasis	8(13.1)
Liver metastases	3(4.9)
Bone metastases	3(4.9)

### 3.2 Treatment efficacy

Short-term efficacy outcomes are presented in **Table 2**. Among the 61 patients with measurable lesions, 28 (45.9%) achieved a partial response and 24 (39.3%) had stable disease, yielding an ORR of 45.9% and a DCR of 85.2%.

**Table 2:** Treatment response assessed per RECIST version 1.1.

Patient with measurable lesions	N = 61 (%)
CR	0 (0)
PR	28 (45.9)
SD	24 (39.3)
PD	9 (14.8)
ORR	28 (45.9)
DCR	52 (85.2)

### 3.3 Survival outcomes

**Figure 2:**
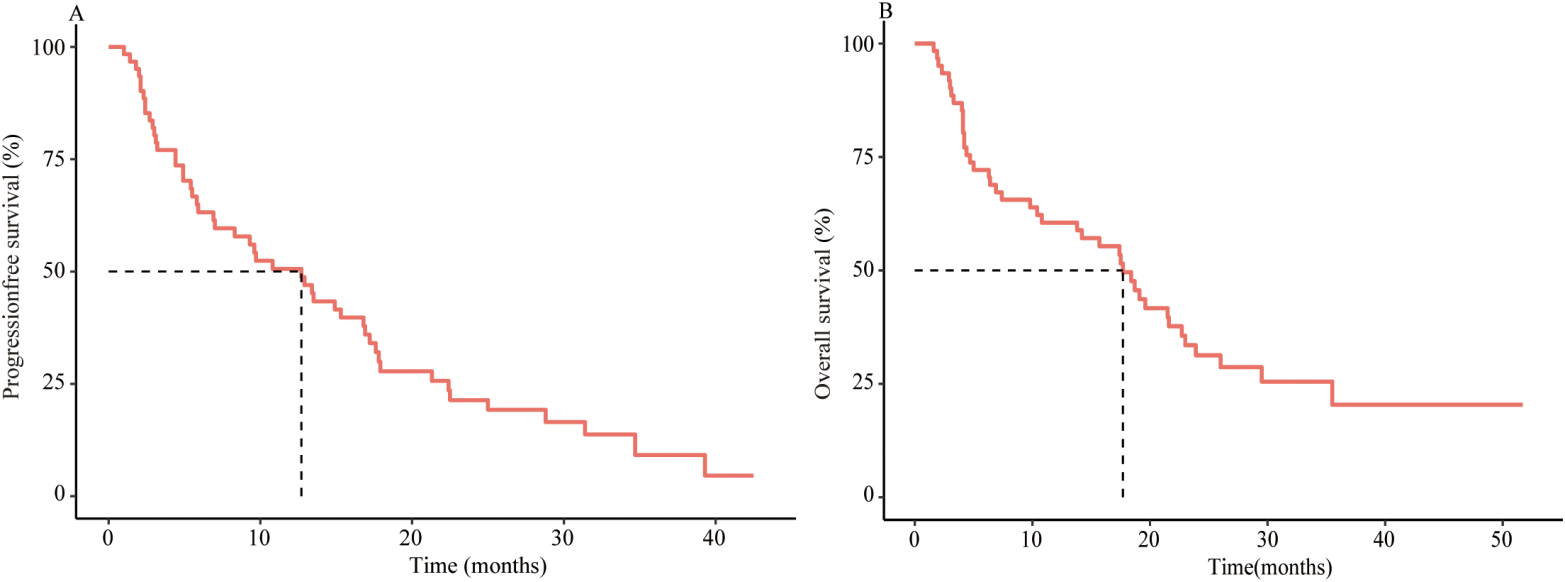
Survival analysis of first-line immunotherapy treatment in patients aged ≥75 years with Advanced ESCC. Kaplan–Meier analysis of PFS **(A)** and OS **(B)** in 61 patients aged ≥75 years with Advanced ESCC treated with first line immunotherapy.

After a median follow-up of 16.7 months (range: 1.6-51.7), the median PFS for the entire cohort was 12.7 months (95% CI: 6.9–17.1), and the median OS was 17.7 months (95% CI: 10.7–22.8) (**Figures 2A, B**).

**Figure 3:**
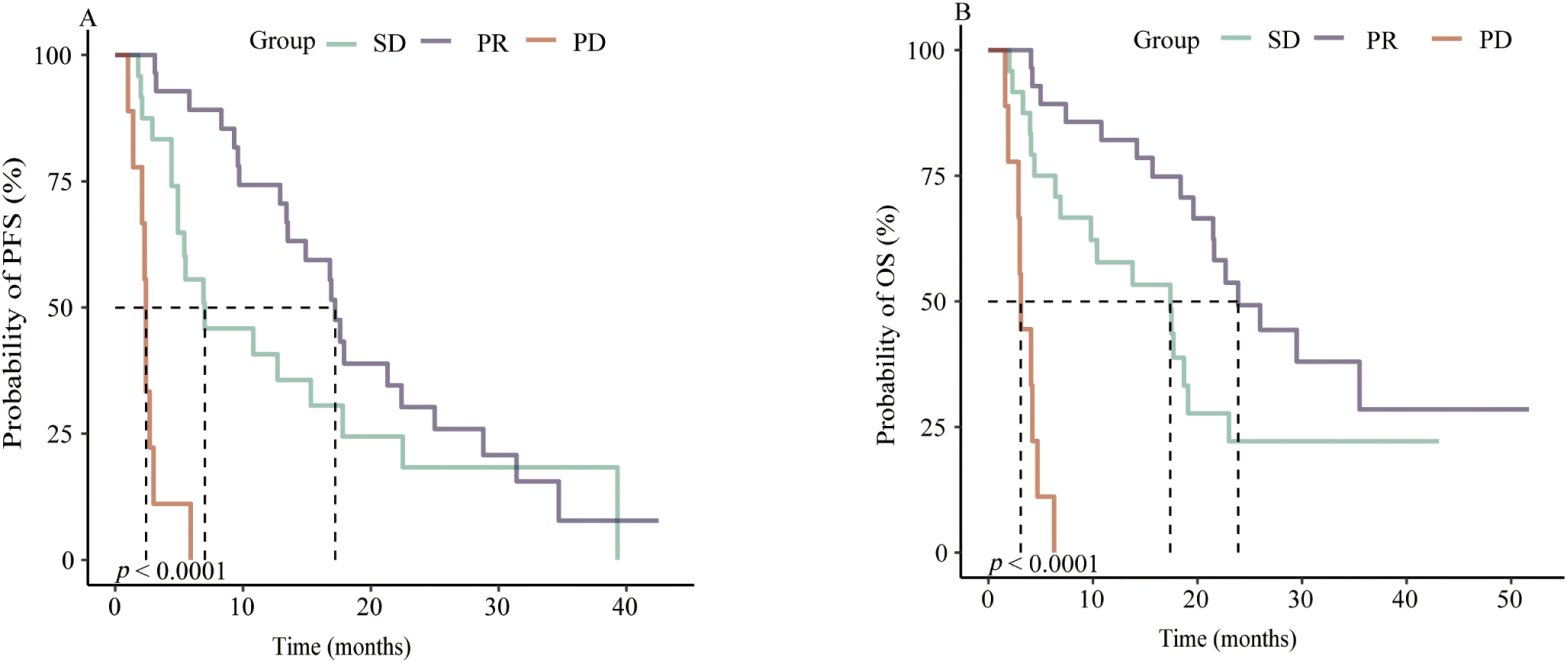
Kaplan–Meier analysis of PFS **(A)** and OS **(B)** in 61 patients aged ≥75 years with Advanced ESCC treated with first line immunotherapy according to response subgroup.

The cohort was stratified according to best overall response into stable disease (SD, n = 24), partial response (PR, n = 28), and progressive disease (PD, n = 9). Both PFS and OS differed significantly among these groups (log-rank *p* < 0.0001 for both) (**Figures 3A, B**).

### 3.4 Factors associated with PFS and OS

Based on the univariate and multivariate analyses presented in **Tables 3**, **4**, several prognostic factors for PFS and OS were identified in this cohort of patients aged ≥75 years with advanced ESCC treated with first-line immunotherapy.

**Table 3:** Univariate analysis identifying prognostic factors for PFS and OS in the entire cohort.

Characteristics		PFS	*P* value	OS	*P* value
		HR (95% CI)		HR (95% CI)	
Gender	Male	Ref		Ref	
	Female	0.94 (0.53,1.66)	0.833	0.56 (0.3,1.05)	0.072
Age	≤80	Ref		Ref	
	>80	1.29 (0.72,2.31)	0.384	1.35 (0.72,2.52)	0.354
CCI	0	Ref		Ref	
	1-2	0.92 (0.43,1.98)	0.836	0.87 (0.38,1.97)	0.737
ECOG -PS	0-1	Ref		Ref	
	2-3	2.79 (1.51,5.15)	0.001	2.37 (1.24,4.53)	0.009
TNM Stage	III	Ref		Ref	
	IV	0.88 (0.5,1.54)	0.643	0.78 (0.42,1.43)	0.416
Radiotherapy	No	Ref		Ref	
	Yes	0.73 (0.41,1.31)	0.293	0.58 (0.31,1.11)	0.098
Immunotherapy	ICI+Chemotherapy	Ref		Ref	
protocols	ICI	4.86 (2.04,11.61)	<0.001	2.91 (1.22,6.94)	0.016

**Table 4:** Multivariate analysis identifying prognostic factors for PFS and OS in the entire cohort.

Characteristics		PFS	*P* value	OS	*P* value
		HR (95% CI)		HR (95% CI)	
ECOG -PS	0-1	Ref		Ref	
	2-3	3.46 (1.56,7.68)	0.002	2.79 (1.24,6.32)	0.014
Immunotherapy	ICI + Chemotherapy	Ref		Ref	
protocols	ICI	5.14 (1.85,14.27)	0.002	3.63 (1.3,10.19)	0.014

In the univariate analysis, poorer performance status (ECOG-PS 2-3) and immunotherapy with ICI monotherapy (compared to ICI combined with chemotherapy) were significantly associated with worse PFS and OS (all *p* < 0.05). Specifically, patients with ECOG-PS 2–3 had a nearly threefold increased risk of progression (HR 2.79, 95% CI 1.51-5.15) and death (HR 2.37, 95% CI 1.24-4.53). Similarly, those receiving ICI alone showed a substantially higher risk for both PFS (HR 4.86, 95% CI 2.04-11.61) and OS (HR 2.91, 95% CI 1.22-6.94) compared to the ICI-chemotherapy combination. Other factors, including gender, age, CCI, Tumor-Node-Metastasis (TNM) stage, and radiotherapy, did not demonstrate statistically significant associations with survival outcomes in univariate analysis.

The multivariate analysis, adjusted for sex, age, TNM stage, and radiotherapy, confirmed ECOG-PS and immunotherapy regimen as independent prognostic factors. ECOG-PS 2–3 remained significantly associated with inferior PFS (HR 3.46, 95% CI 1.56-7.68) and OS (HR 2.79, 95% CI 1.24-6.32). Likewise, ICI monotherapy continued to indicate a higher risk for both PFS (HR 5.14, 95% CI 1.85-14.27) and OS (HR 3.63, 95% CI 1.30-10.19) compared to combination therapy.

### 3.5 Adverse events

The spectrum of adverse events is detailed in **Table 5**. Immune-related adverse events (irAEs) of any grade occurred in 39.1% of patients, with reactive capillary endothelial proliferation observed in 9 patients (14.8%), and grade ≥3 irAEs in 1.6%. Any-grade non-irAEs occurred in 93.1% of patients, including leukopenia in 31 (50.8%), anemia in 6 (9.8%), thrombocytopenia in 3 (4.9%), fatigue in 8 (13.1%), vomiting in 2 (3.2%), nausea in 3 (4.9%), radiation pneumonitis in 2 (3.2%), radiation esophagitis in 1 (1.6%), and tracheoesophageal fistula in 1 (1.6%). Grade ≥3 non-irAEs occurred in 14.7% of patients, including leukopenia in 5 (8.2%), fatigue in 3 (4.9%), and tracheoesophageal fistula in 1 (1.6%).

**Table 5:** Adverse events [n (%)].

irAEs	Grade 1/2 No. (%)	Grade 3/4 No. (%)
Pneumonia	1 (1.6)	0(0)
Myasthenia gravis	1 (1.6)	1(1.6)
Enteritis	2 (3.2)	0(0)
Hepatitis	1 (1.6)	0(0)
Hypothyroidism	5 (8.2)	0(0)
Hyperthyroidism	2 (3.2)	0(0)
Rash	3 (4.9)	0(0)
RCCEP	9 (14.8)	0(0)

Non-irAEs	Grade 1/2 No. (%)	Grade 3/4 No. (%)
Leukopenia	31 (50.8)	5(8.2)
Anemia	6 (9.8)	0(0)
Thrombocytopenia	3 (4.9)	0(0)
Fatigue	8 (13.1)	3(4.9)
Vomiting	2 (3.2)	0(0)
Nausea	3 (4.9)	0(0)
Radiation pneumonitis	2 (3.2)	0(0)
Radiation esophagitis	1 (1.6)	0(0)
Tracheoesophageal fistula	1(1.6)	1(1.6)

RCCEP, Reactive cutaneous capillary endothelia.

## 4 Discussion

This retrospective, real-world study provides one of the first focused evaluations of first-line immune checkpoint inhibitors (ICIs) in patients aged ≥75 years with advanced ESCC, a population systematically underrepresented in clinical trials. We observed promising efficacy-ORR of 45.9%, DCR of 85.2%, mPFS of 12.7 months, and mOS of 17.7 months-coupled with a manageable safety profile. These findings provide valuable preliminary evidence to guide clinical decision-making for oncologists managing this growing and clinically complex elderly population.

The efficacy outcomes in our cohort appear generally consistent with the survival benefits demonstrated for ICI-chemotherapy combinations in pivotal global phase 3 trials, such as KEYNOTE-590 (10), ESCORT-1st (9), ORIENT-15 (12), and RATIONALE-306 (13), which established the current first-line standard. However, direct comparisons are inherently limited. Those trials primarily enrolled younger, fitter patients and often excluded or underrepresented the very elderly (≥75 years). Furthermore, our real-world cohort exhibited therapeutic heterogeneity, with most (85.3%) receiving ICI-chemotherapy but a minority receiving ICI monotherapy (13.1%), reflecting individualized clinical judgment.

In our analysis, baseline functional status (ECOG) and initial immunotherapy strategy emerged as dominant independent prognostic factors. Patients with an ECOG performance status of 2–3 had nearly a threefold higher risk of disease progression and death compared to those with a status of 0–1. This finding reinforces that functional status, a key surrogate for physiological reserve and frailty, remains a paramount prognostic and predictive factor in geriatric oncology, often outweighing chronological age alone (14, 16, 21). The significantly inferior outcomes associated with ICI monotherapy compared to ICI–chemotherapy (adjusted hazard ratio for progression-free survival: 5.14) are consistent with level-1 evidence supporting combination therapy (9–12). Mechanistically, cytotoxic chemotherapy may promote immunogenic cell death and modulate the tumor microenvironment to potentiate ICI activity (17). However, in this observational study, confounding by indication is a critical consideration; clinicians likely selected monotherapy for frailer patients or those with comorbidities contraindicating chemotherapy, which may bias the results in favor of combination regimens.

The safety profile in our cohort warrants nuanced interpretation. Serious irAEs were infrequent (grade ≥3: 1.6%), consistent with aggregate evidence that the incidence of high-grade irAEs does not appear markedly increased in older patients treated with immune checkpoint inhibitors (ICIs) compared to younger cohorts (14, 17). Meanwhile, chemotherapy-related hematologic toxicity was common and clinically meaningful, with leukopenia in over half of patients and grade ≥3 non-immune events in 14.7%. This finding underscored that the tolerability of combined ICI-chemotherapy in elderly patients was driven largely by the chemotherapy component rather than by immunotherapy, a pattern reported in other real-world and subgroup analyses (14, 15, 17). Therefore, the combination of ICIs and chemotherapy in older adults should involve a judicious, individualized selection of cytotoxic agents, with close attention to dose intensity and the proactive integration of supportive care measures. A substantial minority (41%) of patients received radiotherapy during the treatment course. While combined-modality therapy may enhance local control and occasionally synergize with systemic immunotherapy, it also introduces potential additive toxicity. In our cohort, serious local complications were observed, including tracheoesophageal fistula, as well as radiation-related pneumonitis and esophagitis. Published reports and case series have raised concerns about an increased risk of esophageal perforation or fistula when radiotherapy is combined with anti–PD-1/PD-L1 agents in patients with locally advanced esophageal tumors, particularly in the presence of re-irradiation, tumor necrosis, prior stenting, or tumor invasion of the airway or esophagus (22–24). These observations highlight the need for multidisciplinary risk assessment and careful attention to timing, dose planning, and follow-up when integrating radiotherapy with ICIs in elderly patients.

The broader implication of our study is that chronological age should not serve as the sole criterion for withholding immunotherapy. A personalized approach, guided by functional status, comprehensive geriatric assessment, and patient preferences, is essential. Our data contribute to the growing body of evidence that selected older adults with advanced cancer can derive meaningful clinical benefit from ICIs.

We explicitly acknowledge several important limitations of our study. The retrospective, single-center design and the absence of a control group limit the ability to draw causal inferences and may introduce potential selection and center-specific biases. Treatment heterogeneity-both in systemic regimens and in the use of radiotherapy (administered in 41% of patients)-while reflective of real-world clinical practice, complicates the isolation of the ICI effect. The modest sample size (N = 61), particularly the small progressive disease subgroup (n = 9), restricts statistical power for subgroup analyses. The lack of PD-L1 expression and other biomarker data represents a significant gap, as these are established predictive markers in ESCC. Despite these limitations, research in this underrepresented population remains of high clinical value. Future prospective, multicenter studies with larger sample sizes, integrated geriatric assessments, and biomarker profiling are urgently needed to validate these findings and refine optimal treatment strategies for the growing population of older adults with advanced ESCC.

## 5 Conclusions

Our study provides real-world evidence that first-line immunotherapy offers promising efficacy and a manageable safety profile in patients aged ≥75 years with advanced ESCC. These findings may help guide clinical decision-making for this vulnerable population. Future prospective studies with larger sample sizes and integration of biomarkers are warranted to validate these results and identify optimal treatment strategies.

## Data Availability

The raw data supporting the conclusions of this article will be made available by the authors, without undue reservation.
